# Feasibility and safety of antepartum tactile imaging

**DOI:** 10.1007/s00192-020-04552-6

**Published:** 2020-10-17

**Authors:** Zdenek Rusavy, Vladimir Kalis, Salavat Aglyamov, Vladimir Egorov

**Affiliations:** 1 Department of Obstetrics and Gynecology, Faculty of Medicine in Plzen, Charles University, Pilsen, Czech Republic; 2 Biomedical Center, Faculty of Medicine in Plzen, Charles University, Pilsen, Czech Republic; 3 Department of Gynecology and Obstetrics, University Hospital in Pilsen, Pilsen, Czech Republic; 4 University of Houston, Houston, TX USA; 5 Advanced Tactile Imaging, Trenton, NJ USA

**Keywords:** Perineal elasticity, Tactile imaging, Elastography, Finite element model, Biomechanics of parturition

## Abstract

**Introduction and hypothesis:**

Quantitative characterization of the birth canal and critical structures before delivery may provide risk assessment for maternal birth injury. The objective of this study was to explore imaging capability of an antepartum tactile imaging (ATI) probe.

**Methods:**

Twenty randomly selected women older than 21 years with completed 35th week of pregnancy and a premise of vaginal delivery were enrolled in the feasibility study. The biomechanical data were acquired using the ATI probe with a double-curved surface, shaped according to the fetal skull and equipped with 168 tactile sensors and an electromagnetic motion tracking sensor. Software package COMSOL Multiphysics was used for finite element modeling. Subjects were asked for assessment of pain and comfort levels experienced during the ATI examination.

**Results:**

All 20 nulliparous women were successfully examined with the ATI. Mean age was 27.8 ± 4.1 years, BMI 30.7 ± 5.8, and week of pregnancy 38.8 ± 1.4. Biomechanical mapping with the ATI allowed real-time observation of the probe location, applied load to the vaginal walls, and a 3D tactile image composition. The nonlinear finite element model describing the stress–strain relationship of the pelvic tissue was developed and used for calculation of Young’s modulus (E). Average perineal elastic modulus was 11.1 ± 4.3 kPa, levator ani 4.8 ± 2.4 kPa, and symphysis–perineum distance was 30.1 ± 6.9 mm. The pain assessment level for the ATI examination was 2.1 ± 0.8 (scale 1–4); the comfort level was 2.05 ± 0.69 (scale 1–3).

**Conclusions:**

The antepartum examination with the ATI probe allowed measurement of the tissue elasticity and anatomical distances. The pain level was low and the comfort level was comparable with manual palpation.

## Introduction

Childbirth may be associated with trauma, which can have severe consequences and impact on the woman’s health. In the course of a vaginal delivery, significant deformations of pelvic floor soft tissues occur that may damage its integrity. Observational studies from the UK show that merely 9.6% of women have an intact perineum after their first vaginal delivery [1]. Former studies have shown that about 85% of women suffer some degree of perineal injury, with 60–70% requiring a suture [2, 3]. The consequences of perineal tears, such as anal incontinence, severe perineal pain or distal rectocele [4], may be associated with significant morbidity along with a significant impact on the quality of life and healthcare costs. Dyspareunia, urine and stool incontinence and pelvic organ prolapse resulting from levator ani and/or perineal trauma may exclude the women from social life. The presence and severity of these complications usually depends on the extent of the injury to the pelvic floor, i.e., the levator ani and perineum [5]. Given the frequency of birth trauma, any well-implemented and effective method of prevention has a great impact on the quality of life of the whole population. Identifying women at an increased risk of severe perineal or levator ani injury during childbirth remains a key element in targeting prevention and planning health allocation strategies. It is impossible to counsel women regarding trauma and intervene around the time of childbirth without knowledge of the woman’s individual risk. Assessment of the risk of perineal trauma and its consequences is difficult, given multiple risk factors. Among them are biomechanical conditions of the critical structures along the birth canal (perineum and levator ani), fetal size, and bony pelvis size. Several models based on the physical features of the mother and the fetus have been developed to assess the risk of pelvic floor trauma and long-term risk of childbirth complications [6–8]. Nevertheless, these models do not include an important variable, i.e., perineal elasticity and its reversible distensibility from the pubic symphysis. There is significant inter-individual variation in the stress–strain behavior of the perineal tissue and at the same time, it is probably the most important determinant of perineal trauma. Furthermore, there exists no objective method for perineal elasticity assessment with deformations similar to vaginal delivery. Adding this information and taking into account the material properties of the woman’s birth canal would undoubtedly significantly improve the risk assessment quality. Therefore, a reliable method able to objectively measure and evaluate material tissue properties of the birth canal and perineum in a pregnant woman before vaginal birth is required. The objective of this study was to explore the capability of an antepartum tactile imaging (ATI) in perineal stress–strain (elasticity) characterization and measurement of perineum distance from the pubic symphysis.

## Materials and methods

### Antepartum tactile imaging

Tactile imaging is a medical imaging modality translating the sense of touch into a digital image. The tactile image is a function of three spatial coordinates, *P(x,y,z)*, where *P* is the pressure on the soft-tissue surface under applied deformation and *x,y,z* are coordinates where the pressure *P* is measured. The tactile image is a pressure map on which the direction of tissue deformation must be specified [9].

**Fig. 1:**
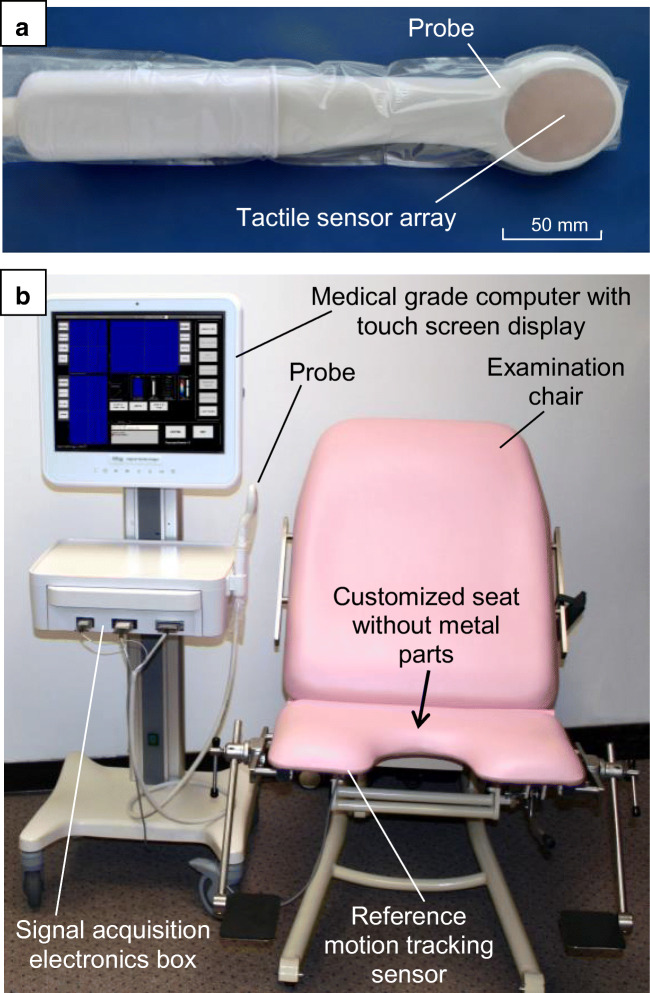
Antepartum tactile imager. **a** Vaginal probe with tactile sensor array and motion tracking sensor. **b** General view of the device

The vaginal tactile imaging probe used in previous studies [10, 11] is not suitable for antepartum evaluation of tissue characteristics of the perineum. Its shape did not allow for the simulation of the perineal deformation occurring during expulsion of the fetal head and it does not have a 3D motion tracking sensor. Therefore, a perineal probe was designed with a double-curved surface, based on the shape of a neonate skull to approximate the shape of the fetal head in the area that is in contact with the perineum. Previously, four design prototypes without incorporated sensors were tested with respect to the ease of insertion, ease of examination, and similarity of perineal deformation with the deformation seen after vaginal delivery. The prototypes were covered with a sterile sheath and lubricated and tested as part of antenatal consultations. This probe design study was also approved by the ethics committee and the participants signed informed consent. Furthermore, women’s discomfort and pain were evaluated and compared with vaginal obstetric examination and transvaginal ultrasound examination (not published). Based on these preliminary results, the new probe with 168 tactile sensors on a double curved surface with a 55-mm radius (transverse) and 90-mm radius (along the probe) with an oval sensing area of 41 mm by 53 mm was designed (Fig. 1a). The probe’s dimensions are 54 mm in width and 68 mm in length. The probe has a six-degrees-of-freedom electromagnetic motion tracking sensor (Polhemus, Colchester, VT, USA), which allows acquisition of 3D coordinates and angles of the probe, relative to a reference motion tracking sensor, placed under the seat of a custom-designed examination chair to eliminate interference for electromagnetic tracking.

The tactile imaging probe was used with a lubricating gel to provide reproducible boundary/contact conditions with the deformed tissue; these conditions are classified as slip boundary conditions. The tactile probe measures an applied pressure, but not force. Force is a vector and by definition has amplitude and direction. The pressure sensors designed for this probe are not sensitive to the tangential component of a force that may arise during probe motion. The sensors measure pressure, defined as the orthogonal component of force divided by area. The probe may be used not only for tissue compression in the orthogonal direction to the tissue surface but also for sliding over the tissue and/or probe elevation. These probe maneuvers allow accumulation of multiple pressure patterns from the tissue surface to compose an integrated tactile image for the region of interest [10].

An ATI device was designed as a cart-based device with a detachable probe, signal acquisition electronics, a medical grade touchscreen computer (Tangent, CA, USA) and the examination chair. Proprietary data processing software was used to calculate perineal elasticity and anatomical distance from the lower margin of the pubic symphysis and perineal surface—the symphysis–perineum critical distance (D) at a local pressure of 20 kPa by the ATI probe if achieved or maximum pressure in the case of an elastic perineum with a low Young’s modulus. The ATI quantified perineal elasticity using Young’s modulus, calculated from spatial gradients in the resulting 3D tactile images, with the use of the developed finite element model (see below).

### Study design

This observational study was performed from August 2019 to December 2019 (clinical trials identifier NCT03883867). It was approved by the Institutional Review Board and written informed consent was obtained from each patient prior to study enrollment.

The inclusion criteria were adult pregnant women, age 21+ years, after completing the 35th week of pregnancy, with the fetus in the vertex position and the premise of vaginal delivery. Exclusion criteria were prior perineal surgery, HIV or hepatitis B-positive serology, warty lesions on the vulva, extensive varicose veins on the vulva, active skin infection or ulceration within the vagina/vulva (Herpes infection), presence of a vaginal septum, severe hemorrhoids, and stillbirth or extensive congenital abnormalities of the fetus.

During the examination, patients were placed in the dorsal lithotomy position. The ATI probe was covered by a disposable plastic sheath with a lubricant. Three orthogonal projections of the 3D vaginal pressure map with real-time ATI probe location were observed by the operator. The examination was performed manually by the operator and iterated 2–3 times in the first 10 cases in order to find the best examination procedure. Once the optimal examination procedure was found, it was performed in all subjects. The maximum average pressure load applied by the probe was 20 kPa. The complete examination lasted for 3–5 min. Downward movement of the probe while compressing the tissues and sliding the probe along the vaginal canal were used for the 3D tactile image composition. Patients were queried regarding pain and comfort after the examination. The pain was described using a four-point scale with 1 no pain, 2 mild pain, 3 moderate pain, and 4 severe pain. The comfort level was assessed using a second three-point scale with 1, more comfortable than manual palpation, 2, as comfortable as manual palpation, and 3, less comfortable than manual palpation. Safety was assessed with recorded pain level and an adverse event log.

### Finite element modeling

**Fig. 2:**
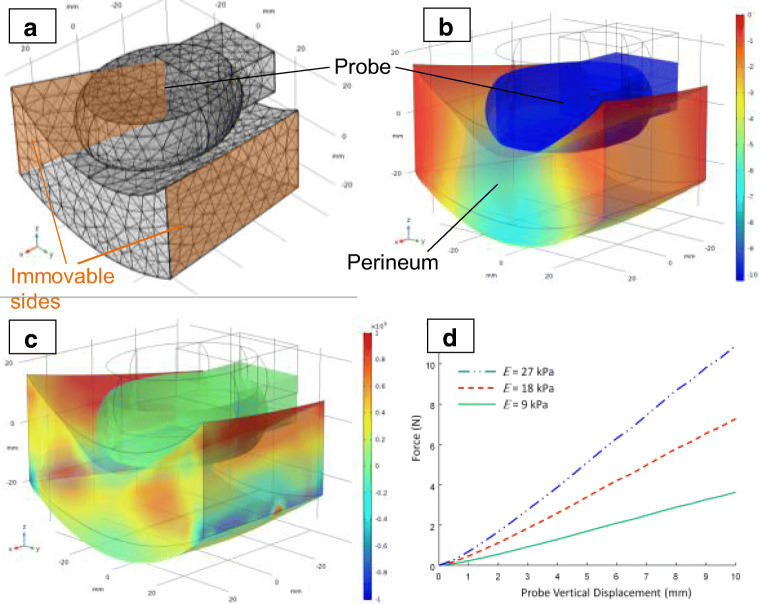
Results of finite element modeling of the antepartum tactile imaging probe interaction with the perineum. **a** 3D model geometry. **b** 3D distribution of vertical displacement in the perineum. **c** 3D distribution of vertical stress in the perineum. **d** Force applied to the probe versus vertical displacement for various Young’s moduli of the perineum

The finite element model (FEM) of the perineal deformation by the ATI probe was developed using a commercial software package, COMSOL Multiphysics (COMSOL, Stockholm, Sweden). The probe geometry in the model simulated the real ATI probe geometry. The contact between the probe and the top surface of the tissue layer was considered to be frictionless to simulate the probe lubrication during studies. Stress-free boundary conditions of soft tissues were assumed for all other surfaces, except for the immovable sides, as shown in Fig. 2a, for which the fixed conditions were assumed to simulate connection to the bones. The nonlinear elastic properties of the pelvic floor were described with a hyper-elastic Neo-Hookean model with Poisson’s ratio close to 0.5 [12, 13]. The birth canal, including the perineum and the levator ani muscle, was modeled as an incompressible, hyper-elastic layer. Overall, about 140,000 of the tetrahedral elements were used for each calculation.

**Fig. 3:**
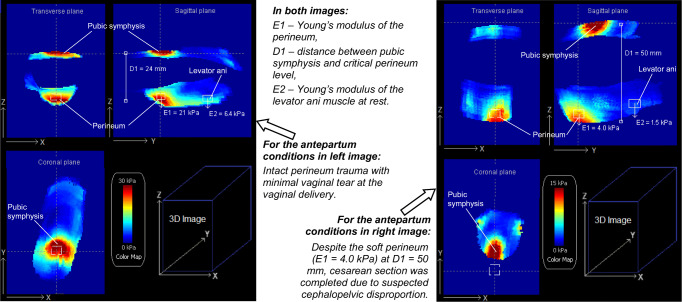
Tactile image. Three orthogonal projections for acquired tactile image demonstrating symphysis–perineum distance (*D*) and perineal elastic modulus (*E*) in two subjects with significant differences in anatomy and biomechanical parameters

The Young’s modulus of the soft tissues was calculated using the FEM results. Specifically, this FEM provides pressure distribution on the surface of the deformed tissue (Fig. 2c) as a function of vertical displacement of the tissue surface (Fig. 2b) with the ATI probe. In other words, the FEM creates a 3D tactile image for the tissue deformations with the ATI probe. The variable of FEM is Young’s modulus of the tissue. FEM simulations have provided a set of functions *F(z)*, where *F* is the force applied to the probe or to the tissue surface and *z* is the vertical probe or tissue surface displacement. For illustrative purposes, Fig. 2d shows three functions of *F(z)* for specified values of Young’s modulus (*E*). The slopes *dF(z)/dz* were used for calculation of *E* for the soft tissues within the acquired 3D tactile images (Fig. 3). These calculations must be understood to be approximations. The described FEM itself is the approximation of real complexities. However, the experimental validation of similar model strain–stress calculations allowed accuracy of ±10–15% for Young’s modulus measuring with silicone models in the E range of 2 kPa to 20 kPa [10].

## Results

All 20 enrolled women were successfully examined with the ATI. Average age was 27.8 ± 4.1 years, BMI 30.7 ± 5.8, week of pregnancy 38.8 ± 1.4. All women were nulliparous. Seventeen of the 20 women had vaginal delivery (10 spontaneous, 5 induced, and 2 vacuum-assisted) and 3 had a cesarean section. One woman sustained anal sphincter injury during her vaginal delivery, despite a mediolateral episiotomy.

The developed examination procedure consisted of inserting the probe into the vagina and obtaining a contact with the perineal body, applying pressure to the posterior vaginal wall while slowly sliding the probe to the vaginal introitus, gradually applying load to the posterior vaginal wall and the perineum, returning the probe above the levator plate, and rotating the probe 180° with a minimal load to the vaginal walls, moving the probe along the anterior vaginal wall and applying load to capture tactile signal from the pubic symphysis. The primary targeted structures for the tactile imaging were the perineum, levator ani, and pubic symphysis location.

An example of the numerical simulations for Young’s modulus value of 9 kPa and the layer thickness of 29 mm is presented in Fig. 2, where vertical tissue displacement (Fig. 2b), stress distribution (Fig. 2c), 3D model geometry, and ATI probe (Fig. 2a) are shown. The overall force reaction applied to the probe as a function of the vertical displacement of the probe for three Young’s modulus values are shown in Fig. 2d. The mean perineal elastic modulus was E = 11.1 ± 4.3 kPa, levator ani elasticity E = 4.8 ± 2.4 kPa, and the symphysis–perineum distance at a load of 20 kPa was 30.1 ± 6.9 mm. Owing to the small sample size, no statistically significant clinical correlations between the delivery outcome and examination results could be made. However, significant inter-individual variability was observed with some correlation with the extent of perineal trauma. Figure 3 demonstrates variations in anatomical measure (D) and perineal elasticity (E) between two subjects with significant differences in anatomy and biomechanical parameters.

The pain assessment level for the ATI examination according to the four-point scale was 2.1 ± 0.8; the comfort level was 2.05 ± 0.69. Rotation of the probe was perceived as the most uncomfortable part of the examination procedure. There were no adverse events or complications related to use of the ATI. No harm or trauma to the pregnant woman or the fetus occurred during the examination. No effect on the course of the pregnancy was observed.

## Discussion

In the present study, the new device for the assessment of perineal elasticity in pregnant women was clinically tested. The antepartum tactile imager was proven safe and achieved the examination in all enrolled subjects with discomfort comparable with vaginal examination. The encouraging clinical results were also received in a parallel clinical study, with the ATI probe having s smaller form factor and orthogonal tactile array of 128 sensors with 10 nonpregnant and 10 parous pregnant women [14]. These pioneering results with the ATI probes laid down a foundation for the future development and understanding of the biomechanical characteristics of the birth canal. The in vivo assessment enabled by this device allows examination of an individual woman’s perineal stiffness. The potential of ATI lies in enabling tailored treatment in childbirth trauma prevention. As vaginal delivery is a biomechanical process, it is likely that analysis of a 3D stress–strain of the individual’s perineum using the ATI, together with fetal and other maternal factors could contribute to the calculation of the individual risk of severe childbirth trauma and prediction of how applied procedures and delivery mode may mitigate these risks.

Examination by palpation is a common method that has proven most effective throughout centuries. The tactile sensation allows distinction of structural changes in soft tissues that is typical of some diseases and assessment of tissue elasticity. The problem with manual palpation, however, is that it cannot be quantified, recorded, and reproduced, especially between inexperienced examiners. A reliable method able to objectively measure and evaluate material tissue properties (elasticity) has been under development since the end of the twentieth century. Various methods of ultrasound elastography have been described; some found limited use in clinical practice [15–17]. These methods are not suitable for assessment of birth canal elasticity as they allow for measurement of only a limited area/volume of the soft tissue, without a load applied to tissues comparable with the passage of the fetus through the birth canal. Magnetic resonance elastography is another method of assessing in vivo tissue properties; however, it is not useful for the visualization and assessment of the perineum [9]. Tactile imaging is a medical imaging modality that transfers palpation onto a digital image. The instrument uses the information on the precise change in location of the probe and pressure exerted on the probe during its manipulation. The resulting tactile image is a pressure map with a specified direction of tissue deformation [10, 18]. The mechanism of action is thus similar to human fingers, which deform the examined tissue during the examination and sense their resistance. This method has been proven effective in the examination of the female pelvic floor in urogynecology [11].

Computational models describing deformations of the levator ani muscle during vaginal birth have been developed [19–22]. Although the existing models provide an insight into parturition, they are restricted by numerous assumptions and predominantly by the fact that they are limited to levator ani muscle. Behavior of the perineum during delivery was modeled in only a few studies [23–25]. Although most important for the simulation of preventive strategies for perineal trauma, it is infrequently modeled for the absence of knowledge of in vivo material characteristics of the perineum. The models are often based on the data collected on animal, post-menopausal or cadaver tissues with different properties to a pregnant woman, which represents a potential source of significant inaccuracy in modeling of in vivo deformation [26–28]. Some of these models use simplified boundary conditions and do not always consider the mechanical interaction between the fetal head and maternal pelvic tissues. Thus, to date, we cannot find a validated in vivo biomechanical model of vaginal delivery that could describe the pelvic tissue behavior under significant stress and predict maternal injury of pelvic soft tissues. In this study, the nonlinear hyper-elastic Neo-Hookean model was used. Although more complex nonlinear models could describe tissue mechanical behavior more accurately, they require additional elastic constants. The Neo-Hookean model of incompressible medium requires only one elastic constant, Young’s modulus or shear modulus, to describe the stress–strain relation in tissue. Further development and bench/clinical validation will allow optimization of the biomechanical description of the pelvic floor tissues.

Also, more research is necessary before ATI could become a valuable tool. If proven effective, the device would be practical in prenatal counseling. We would be able to show an individual woman how elastic her perineum is. We could calculate/estimate her risk of severe childbirth trauma and counsel her regarding possibilities available making the perineum more elastic. The progress and effectiveness of the suggested methods could also be monitored. Perineal massage has been proven effective in severe childbirth trauma prevention [29, 30]. However, the compliance and motivation of the patients are paramount to its success. The knowledge of individual perineal elasticity and its change over time after the massage could be an important motivation factor. Women concerned about their childbirth trauma having high perineal elasticity could be assured and supported to deliver vaginally. Furthermore, the device could be very useful in understanding the tissue changes that occur during many physiological states. Does perineal stiffness decrease during pregnancy? Is this decrease gradual or very quick near delivery? Is perineal stiffness permanently altered after a first vaginal delivery?

The strength of this study is the development of the new probe design and adapted examination procedure, and the fact that this is the first report on the measurements of perineal elasticity in nulliparous pregnant women with the oval double-curved tactile sensors array. The data obtained have the potential to increase the reliability of finite element modeling of the pelvic floor, specifically the perineum.

A limitation of this study is the fact that all measurements in this study were performed by a single examiner at one center and the number of participants is fairly low. The small sample size has a limited power to evaluate the association between perineal elasticity and symphysis–perineum distance with maternal birth injuries. The intention of this study, however, was not to assess clinical outcomes and ATI efficacy, but rather to assess the safety and feasibility of the ATI examination.

## Conclusions

A novel probe for antepartum perineal elasticity assessment in pregnant women was designed and clinically tested for safety and feasibility. The examination procedure was established. The produced 3D tactile image of the pelvic floor allowed measurements of the anatomical distances and tissue/structure elasticity. The pain level was low with comfort level comparable with manual palpation. The quantitative characterization of the vaginal birth canal and critical structures have the potential for carrying out a risk assessment of maternal birth injury before delivery. The ATI could be another way of individualizing the approach to pregnant women and of making them more involved/motivated in the prevention of their severe childbirth injury.
